# Distinct financial and social hardships among gastrointestinal cancer survivors: a national survey analysis

**DOI:** 10.1007/s11764-026-02004-6

**Published:** 2026-03-18

**Authors:** Meher Angez, Odysseas P. Chatzipanagiotou, Areesh Mevawalla, Azza Sarfraz, Elemosho Abdulaziz, Qaidar Alizai, Timothy M. Pawlik

**Affiliations:** Department of Surgery, The Ohio State University Wexner Medical Center and James Comprehensive Cancer Center, 395 W. 12Th Ave., Suite 670, Columbus, OH USA

**Keywords:** Financial hardship, Social hardship, Gastrointestinal

## Abstract

**Background:**

High and persistent costs of cancer care often burden survivors, leading to medical financial hardship and, in turn, unmet health‑related social needs (HRSNs) that may hinder treatment adherence and quality-of-life. Gastrointestinal (GI) cancers, often linked with high symptom burden and intensive therapies, may disproportionately aggravate such hardships. We sought to assess medical financial hardship, HRSNs and the interplay of these factors among cancer survivors compared with the general US population.

**Methods:**

The National Health Interview Survey (2013–2018) was used to identify adults (≥ 18 years) categorized as GI cancer survivors, non‑GI cancer survivors, and cancer‑free controls. Medical financial hardship was defined as difficulty paying medical bills, cost-related worry, or delayed/forgone care. HRSNs included food insecurity, housing insecurity and transportation barriers. Survey‑weighted logistic regression was utilized to estimate adjusted prevalences and odds ratios (ORs).

**Results:**

Among 190,113 adults, 1,722 (0.9%) were GI and 12,264 (6.5%) were non‑GI cancer survivors. Cancer survivors were more likely to have household income ≤ 138% of the poverty guideline (GI: 21.1%; non-GI: 16.7%; controls: 19.9%) and less likely to be uninsured (GI: 4.5%; non-GI: 5.6%; controls: 13.6%) than the general population (both p < 0.001). Among cancer survivors with medical financial hardship, the adjusted prevalence of food insecurity (18.7% vs. 15.6%), housing insecurity (36.7% vs. 33.5%), any HRSN (43.7% vs. 39.5%) and multiple HRSNs (13.6% vs. 11.9%) were higher among GI versus non-GI cancer survivors (all p < 0.001). GI cancer survivors with financial hardship were more likely to experience food (21.0% vs. 4.8%), housing (35.6% vs. 11.7%) and transportation insecurity (5.0% vs. 1.9%) (all p < 0.05). GI cancer survivors had higher odds of food (aOR 1.69, 95%CI 1.31–2.20) and housing insecurity (aOR 1.35, 95% CI 1.10–1.66), as well as multiple HRSNs (aOR 1.80, 95%CI 1.36–2.39) compared with the general population; this effect was mitigated among non-GI cancer survivors (food insecurity: aOR 1.22; any HRSN: aOR 1.09; multiple HRSNs: aOR 1.36; housing insecurity: p = 0.181).

**Conclusion:**

Cancer survivors, particularly individuals with GI cancer, faced greater financial hardship and were more likely to be burdened by HRSNs. Routine screening and targeted interventions are warranted for all cancer survivors, with even greater focus on financially burdened GI cancer survivors.

**Implications for Cancer Survivor:**

Routine screening and targeted interventions are warranted for all cancer survivors, with even greater focus on financially burdened GI cancer survivors.

**Supplementary Information:**

The online version contains supplementary material available at https://doi.org/10.1007/s11764-026-02004-6.

## Introduction

Cancer survivors in the United States frequently face medical financial hardship due to the high and ongoing costs of care [1, 2]. More than one-third of patients report some form of hardship, manifesting as material challenges (e.g., unpaid medical bills), psychological strain (e.g., cost-related worry), or behavioral changes (e.g., delaying or forgoing care) [1–5]. These burdens extend beyond economic stress, as financial hardship has been associated with higher rates of anxiety, emergency visits, hospitalizations, and even increased mortality [1, 5]. With the cancer survivor population projected to exceed 20 million in the near future, the public health implications of financial hardship represent a significant issue.

Health-related social needs (HRSNs) such as food insecurity, unstable housing, and transportation barriers, are increasingly recognized as key determinants of cancer outcomes [6]. Of note, financial strain and unmet social needs frequently co-occur with more than half of survivors reporting financial hardship also having at least one HRSN [7, 8]. Food insecurity alone affects nearly one in ten survivors, while housing and transportation barriers further contribute to treatment non-adherence and poor health [9]. Importantly, cancer survivors reporting both financial hardship and HRSNs face markedly greater risks of acute care utilization and mortality [10–14]. Yet, evidence about survivor subgroup disparities remains limited. Specifically, gastrointestinal (GI) cancer survivors may be particularly vulnerable as their treatment often involves complex operative procedures and multimodal therapy leading to disability and frequent disruptions in employment and income [14, 15].

The objective of the current study was to examine the intersection of medical financial hardship and HRSNs among cancer survivors using data from the National Health Interview Survey (NHIS). Specifically, GI cancer survivors were compared with non-GI survivors and adults without a cancer history focusing on three modifiable HRSNs—food insecurity, housing insecurity, and transportation barriers. We sought to identify possible disparities across different cohorts of individuals to inform survivorship care strategies such as financial navigation, social risk screening, and resource linkage for individuals at greatest risk.

## Methods

### Data source and study population

Data from the NHIS, a nationally representative, cross-sectional household survey of the civilian, noninstitutionalized U.S. population, were queried for calendar years 2013 through 2018 via the Integrated Public Use Microdata Series (IPUMS) platform. The NHIS uses a complex, multistage, stratified, clustered sampling design to ensure geographic diversity and population representativeness. Eligible respondents were adults aged 18 years or older who self-reported a history of cancer on the NHIS Sample Adult questionnaire and completed survey modules addressing medical financial hardship (MFH), food insecurity, housing insecurity, and transportation barriers (Supplementary Figure S1). Records with missing data for the exposure, outcomes, or core covariates (age, sex, race/ethnicity, education, marital status, household income, insurance status, or region) were excluded from the multivariable analyses. Participants were categorized into one of three mutually exclusive cohorts: survivors of GI cancers (i.e., colorectal, pancreatic, liver/gallbladder, gastric, or esophageal), survivors of all non-GI cancers, and a control group consisting of the general adult population without cancer history.

### Exposures, covariates, and outcomes of interest

The primary exposure was medical financial hardship within the preceding 12 months, defined by the presence of at least one of the following factors: difficulty paying medical bills or carrying medical debt, current worry about medical costs, or delaying/forgoing medical care due to cost [12]. Five HRSN-related outcomes were assessed: (1) food insecurity, defined as a USDA 10-item food security module score ≥ 3 in the past 30 days; (2) housing insecurity, defined as self-reported worry about paying rent or mortgage; (3) transportation barriers, defined as delayed or forgone medical care in the past year due to lack of transportation; (4) any HRSN, defined as the presence of at least one of the above three needs; and (5) multiple HRSNs, defined as the concurrent presence of two or more needs [12]. Covariates included demographic factors (i.e. age category, sex, race/ethnicity, educational attainment, marital status), socioeconomic measures (i.e. household income-to-poverty guideline ratio (NHIS imputed), health insurance status, and geographic region consistent with NHIS analytic standards.

### Statistical analysis

Categorical variables were reported as frequencies with proportions (%) and were compared using the Survey-weighted Rao–Scott χ^2^ tests. All analyses incorporated NHIS sampling weights, strata, and primary sampling units to account for the survey’s complex design and to produce nationally representative estimates. Participant characteristics and exposure distributions were described using unweighted sample counts and weighted prevalence estimates with standard errors. Differences across cohorts were evaluated using design-adjusted F tests. The joint distribution of MFH and each HRSN outcome was examined by calculating survey-weighted proportions and associated p-values. Separate survey-weighted multivariable logistic regression models were fit for each binary HRSN outcome, with MFH as the primary independent variable and adjustment for all covariates including age group, sex, race/ethnicity, education level, marital status, household income category, health insurance status, and geographic region. Results were reported as adjusted odds ratios (aORs) and 95% confidence intervals (CIs). Effect modification by household income and additional stratified analyses were conducted by age and cancer type. All hypothesis tests were two-sided, and p < 0.05 was considered statistically significant. We report unweighted sample counts and survey-weighted percentages to produce nationally representative estimates. Analyses were performed using Stata version 18.0 (StataCorp, College Station, TX).

## Results

### Basic sociodemographic characteristics

Among 190,113 adults in the analytic cohort, 0.9% (n = 1,722) were GI cancer survivors, 6.5% (n = 12,264) were non-GI cancer survivors, and 92.6% (n = 176,127) were cancer-free controls. GI cancer survivors were older (≥ 75 years; GI cancer: 34.8% vs. non-GI cancer: 26.5% vs. controls: 6.6%) and more frequently male (GI cancer: 53.7% vs. non-GI cancer: 38.1% vs. controls: 48.8%) (both p < 0.001). GI cancer survivors were more likely to be Non-Hispanic Black (9.6%, n = 184 vs. 8.0%, n = 1,128) compared with non-GI cancer survivors, but less likely than the control group (12.5%, n = 23,534; p < 0.001; Table 1). Of note, GI cancer survivors more frequently had high school education or less (46.3%, n = 845) compared with non-GI cancer survivors (38.3%, n = 4,829) and controls (37.3%, n = 67,128) (p < 0.001). Furthermore, GI cancer survivors were more likely to have household income ≤ 138% of the poverty guideline (21.1%, n = 453) compared with non-GI cancer survivors (16.7%, n = 2,576) and cancer-free controls (19.9%, n = 42,380) (p < 0.001).

**Table 1:** Sociodemographic characteristics of the analytic cohorts; General U.S. adults, non‑GI cancer survivors, and GI cancer survivors (NHIS 2013–2018)

Characteristic	General US population N = 176,127 (92.64%)	Non-GI cancer survivor N = 12,264 (6.45%)	GI cancer Survivor N = 1,722 (0.91%)	p-value
Age group, years				
18–44	76,775 (48.9)	1,101 (10.2)	82 (7.7)	< 0.001
45–54	29,384 (17.8)	1,262 (11.9)	146 (10.9)	
55–64	30,123 (16.3)	2,609 (23.5)	344 (21.1)	
65–74	23,406 (10.4)	3,632 (28.1)	466 (25.4)	
≥ 75	16,439 (6.6)	3,660 (26.5)	684 (34.8)	
Sex				
Male	80,237 (48.8)	4,435 (38.1)	874 (53.7)	< 0.001
Female	95,890 (51.2)	7,829 (61.9)	848 (46.3)	
Race/ethnicity				
Non-Hispanic White	136,211 (78.2)	10,518 (87.1)	1,415 (83.8)	< 0.001
Non-Hispanic Black	23,534 (12.5)	1,128 (7.97)	184 (9.57)	
Asian	10,334 (6.2)	293 (2.8)	60 (3.4)	
Others/Unknown*	6,048 (3.1)	325 (2.2)	63 (3.2)	
Educational attainment				< 0.001
< High school	23,326 (12.5)	1,628 (12)	327 (16.3)	
High school/GED	43,802 (24.8)	3,201 (26.3)	518 (30.0)	
> High school	108,269 (62.2)	7,394 (61.4)	865 (52.6)	
Others	730 (0.48)	41 (0.40)	12 (1.06)	
Marital status				< 0.001
Married	77,383 (52.4)	5,533 (58.6)	709 (55.3)	
Separated/divorced	29,449 (11.0)	2,558 (15.1)	341 (13.9)	
Widowed	15,460 (5.3)	2,626 (15.0)	453 (18.7)	
Never married/living with partner	53,437 (31.2)	1,531 (11.3)	215 (12.0)	
Others (unknown)	398 (0.16)	16 (0.11)	4 (0.09)	
Household income (% FPL)				< 0.001
≤ 138% FPL	42,380 (19.9)	2,576 (16.7)	453 (21.1)	
139–200% FPL	20,928 (11.2)	1,561 (11.6)	265 (13.9)	
201–400% FPL	50,854 (29.3)	3,758 (30.3)	566 (33.6)	
> 400% FPL	61,965 (39.6)	4,369 (41.3)	438 (31.4)	
Health insurance				< 0.001
Public	22,027 (13.8)	1,426 (14.5)	263 (20.0)	
Private	104,541 (72.6)	6,806 (79.9)	850 (75.5)	
Uninsured	20,734 (13.6)	506 (5.6)	49 (4.5)	
Geographic region				0.0001
Northeast	28,739 (17.6)	2,177 (18.3	309 (20.9)	
Midwest/North Central	38,368 (22.3)	2,853 (23.5)	404 (22.5)	
South	63,122 (36.6)	4,271 (37.1)	621 (36.9)	
West	45,898 (23.5)	2,963 (21.1)	388 (19.6)	

Data are presented as unweighted counts (n) and survey-weighted column percentages (%), unless otherwise noted. The “Other/Unknown*” race/ethnicity category includes American Indian/Alaska Native, multiracial individuals, other unspecified races, and unknown/refused/missing responses. Poverty category was defined using the NHIS imputed income-to-poverty guideline ratio (POVIMPHHS5) and categorized as ≤138%, 139–200%, 201–400%, and >400% of the poverty guideline

### Prevalence of Medical Financial Hardship and Health-Related Social Needs

In total, 642 (38.8%) GI cancer survivors, 4,478 (36.8%) non-GI cancer survivors, and 75,237 (42.9%) controls reported MFH (p < 0.001; Table 2). Housing insecurity was more prevalent among GI cancer survivors (21.1%, n = 339 vs. 18.1%, n = 2,234) compared with non-GI cancer survivors, but less prevalent than in the cancer-free group (22.3%, n = 39,586; p < 0.001). Of note, food insecurity was more common among GI cancer survivors (11.1%, n = 212) compared with both non-GI cancer survivors (8.6%, n = 1,231) and the general population (9.7%, n = 18,787) (p = 0.003). Similarly, GI cancer survivors were the most likely to report transportation barriers to care (GI cancer: 3.1%, n = 70 vs. non-GI cancer: 3.0%, n = 408 vs. cancer-free controls: 1.8%, n = 3,886; p < 0.001). HRSNs clustered among survivors who reported medical financial hardship. Among GI survivors who did versus did not have medical hardship, food insecurity was 21.0% versus 4.8%, housing insecurity 35.6% versus 11.7%, and transport barriers 5.0% versus 1.9%, respectively; among patients with multiple HRSNs (≥ 2) the difference was more pronounced (15.1% vs. 3.2%, respectively) (all p < 0.005). Similar differences were noted among non-GI survivors (any HRSN 41.1% vs 11.8%; multiple HRSNs 13.3% vs. 2.2%) (Table S1).

**Table 2:** Outcomes and the analytic cohorts; General U.S. adults, non‑GI cancer survivors, and GI cancer survivors

Characteristic	General US population N = 176,127 (92.64%)	Non-GI cancer survivors N = 12,264 (6.45%)	GI cancer Survivors N = 1,722 (0.91%)	P Value
Medical financial hardship				< 0.001
No	100,890 (57.1)	7,786 (62.0)	1,080 (61.2)	
Yes	75,237 (42.9)	4,478 (36.8)	642 (38.8)	
Housing Insecurity	39,586 (22.3)	2,234 (18.1)	339 (21.1)	< 0.001
Food Insecurity	18,787 (9.7)	1,231 (8.6)	212 (11.1)	0.003
Transportation barrier to care	3,886 (1.8)	408 (3.0)	70 (3.1)	< 0.001
Any HRSNs	48,277 (26.8)	2,859 (22.6)	451 (26.6)	< 0.001
2 or 3 HRSNs	12,730 (6.4)	886 (6.3)	147 (7.9)	0.144

Data represent survey-weighted estimates. Categorical variables are reported with proportions (%) and continuous variables are reported using median and interquartile range

On multivariable analysis, GI cancer survivors had 69% and 35% higher odds of food insecurity (aOR 1.69, 95%CI 1.31–2.20) and housing insecurity (aOR 1.35, 95%CI 1.10–1.66), respectively, compared with the general population. Additionally, GI cancer survivorship was associated with 41% higher likelihood of experiencing a single HRSN (aOR 1.41, 95%CI 1.17–1.71), and 80% greater odds of reporting multiple HRSNs (aOR 1.80, 95%CI 1.36–2.39) (Table 3). These associations were not as pronounced among non-GI cancer survivors for food insecurity (aOR 1.22, 95%CI 1.10–1.37), housing insecurity (aOR 1.05, 95%CI 0.97–1.14), any HRSN (aOR 1.09, 95%CI 1.02–1.18), and multiple HRSNs (aOR 1.36, 95%CI 1.20–1.53). Non-GI cancer survivorship was associated, however, with 64% greater odds of transportation barriers to care (aOR 1.64, 95% CI 1.37–1.96) compared with cancer-free individuals.

**Table 3:** Multivariable analysis of HRSNs associated with cancer survivor subgroups

Outcome (Reference group: General US population)	Non-GI cancer survivors		GI cancer survivors	
	aOR (95% CI)	p-value	aOR (95% CI)	p-value
Food Insecurity	1.22 (1.10–1.37)	< 0.001	1.69 (1.31- 2.20)	< 0.001
Housing insecurity	1.05 (0.97–1.14)	0.181	1.35 (1.10–1.66)	0.004
Transportation barrier	1.64 (1.37–1.96)	< 0.001	1.18 (0.80–1.74)	0.384
Any HRSN	1.09 (1.02–1.18)	0.013	1.41 (1.17–1.71)	< 0.001
Multiple HRSNs (≥ 2)	1.36 (1.20–1.53)	< 0.001	1.80 (1.36–2.39)	< 0.001

aOR = adjusted odds ratio; HRSN = health−related social need. Multivariable, survey‑weighted logistic regression of each health‑related social need (HRSN) adjusting for age group, sex, race/ethnicity, education, marital status, income, insurance, geographic region, and cancer site. Models are stratified by survivor cohort (non‑GI vs. GI) general US population is the reference and “no hardship” is the reference

### Adjusted odds of social needs stratified by medical financial hardship

GI cancer survivors reporting financial hardship demonstrated greater odds of food insecurity (aOR 1.69, 95%CI 1.22–2.35), housing insecurity (aOR 1.34, 95%CI 1.04–1.73), any HRSN (aOR 1.44, 95%CI 1.12–1.85), and multiple HRSNs (aOR 1.72, 95%CI 1.21–2.46) compared with cancer-free controls. Of note, similar associations were observed among GI cancer survivors without financial hardship relative to food insecurity (aOR 1.70, 95%CI 1.14–2.52), any HRSN (aOR 1.38, 95%CI 1.02–1.88) and multiple HRSNs (aOR 2.01, 95%CI 1.29–3.14) (Table 4).

**Table 4:** Adjusted odds of HRSNs associated with medical financial hardship in cancer survivors

Outcomes (Reference group: US General population)	Medical Financial Hardship Yes		Medical financial Hardship No	
	aOR (95% CI)	P-value	aOR (95% CI)	P-value
Food Insecurity				
GI cancer	1.69 (1.22–2.35)	0.002	1.70 (1.14–2.52)	0.009
Non-GI cancer	1.30 (1.13–1.48)	< 0.001	1.12 (0.94–1.34)	0.194
Housing insecurity				
GI cancer	1.34 (1.04–1.73)	0.021	1.3 (0.97–1.94)	0.071
Non-GI cancer	1.15 (1.04–1.27)	0.004	0.94 (0.82–1.08)	0.407
Transport barrier				
GI cancer	1.11 (0.65–1.90)	0.691	1.29 (0.76–2.19)	0.338
Non-GI cancer	1.70 (1.35–2.14)	< 0.001	1.55 (1.16–2.08)	0.003
Any HRSN				
GI cancer	1.44 (1.12–1.85)	0.004	1.38 (1.02–1.88)	0.037
Non-GI cancer	1.18 (1.07–1.31)	0.001	1.01 (0.89–1.14)	0.822
Multiple HRSNs (≥ 2)				
GI cancer	1.72 (1.21–2.46)	0.002	2.01 (1.29–3.14)	0.002
Non-GI cancer	1.44 (1.25–1.66)	< 0.001	1.19 (0.95–1.49)	0.957

Adjusted odds ratios (aOR) and 95% confidence intervals (CI) for five health‑related social needs (food insecurity, housing insecurity, transportation barrier, any HRSN, and multiple HRSNs ≥2) among GI and non‑GI cancer survivors versus the U.S. general population (reference), stratified by medical financial hardship (Yes/No). Estimates come from survey‑weighted multivariable logistic regression adjusting for age, sex, race, education, marital status, income, insurance, and region. P‑values <0.05 denote statistical significance

In contrast, while non-GI cancer survivors facing financial hardship had greater likelihood of reporting food insecurity (aOR 1.30, 95%CI 1.13–1.48), housing insecurity (aOR 1.15, 95%CI 1.04–1.27), transportation barriers (aOR 1.70, 95%CI 1.35–2.14), any HRSN (aOR 1.18, 95%CI 1.07–1.31), and multiple HRSNs (aOR 1.44, 95%CI 1.25–1.66), there was no association between non-GI cancer survivorship and HRSNs among survivors without financial hardship (Table 4). In models with interaction terms (Supplementary Table S2), significant effects were observed only among non-GI survivors with financial hardship, housing insecurity (aOR 1.48, 95% CI 1.26–1.73; p < 0.001), any HRSN (aOR 1.35, 95% CI 1.15–1.57; p < 0.001), and multiple HRSNs (aOR 1.34, 95% CI 1.04–1.73; p = 0.026). All other interactions including those for GI survivors across outcomes and for food insecurity or transportation barriers among non-GI survivors were not significant (Supplementary Table S2). In age-stratified, survey-weighted multivariable models, the interaction between medical financial hardship and survivor subgroup was generally consistent across age strata (18–64, 65–74, ≥ 75), with no statistically significant interaction for GI survivors across outcomes (Supplementary Table S3).

Additionally, in sensitivity analyses restricted to pancreatic or stomach cancer survivors, medical financial hardship remained associated with any HRSN (OR 3.58, 95% CI 1.05–12.25; p = 0.042), while domain-specific outcomes had directionally similar but less precise estimates due to limited subgroup size and rare events (Supplementary Table S4).

## Discussion

Financial hardship and health-related social needs (HRSNs) including food insecurity and housing instability are increasingly recognized sequelae of cancer care, yet their distribution across survivor subgroups remains not well characterized. Gastrointestinal (GI) cancers often entail intensive, prolonged treatment courses that may magnify economic strain and social vulnerability. Moreover, few studies contextualize survivorship relative to cancer-free adults to assess population-level burden of cancer care. The present study was important because we examined the intersection of financial hardship and unmet social needs across cancer survivorship subgroups, using a cancer-free control group to provide population-level context. Of note, GI cancer survivors carried a substantially greater burden of unmet social needs than both non-GI survivors and individuals without cancer. Specifically, GI survivors had higher odds of food insecurity, housing instability, and multiple concurrent HRSNs, highlighting their vulnerability. In contrast, among patients with a non-GI cancer, the relationship between survivorship and HRSNs were attenuated or absent. Although financial hardship was strongly correlated with HRSNs among all cancer survivors (Table S1), the pattern differed by cancer subtype. Specifically, among non-GI survivors, financial hardship largely explained the presence of unmet needs. In contrast, among GI survivors, the risk of HRSNs persisted even in the absence of medical financial hardship, suggesting that underlying socioeconomic vulnerability, disability, or disease-specific factors may contribute to social risk burden independent of direct medical costs.

Prior studies have demonstrated that cancer survivors with financial hardship are several times more likely to report HRSNs [12]. Similarly, in the current study, nearly one-half of GI cancer survivors who reported financial hardship also disclosed at least one HRSN, a markedly higher prevalence than among individuals without financial hardship. Gaps were wide across all domains based on the presence of financial hardship: food insecurity (21.0% vs. 4.8%), housing instability (35.6% vs. 11.7%) and transportation barriers (5.0% vs. 1.9%). These findings highlight that GI cancer survivors experienced a disproportionate co-occurrence of financial toxicity and basic resource insecurity; a heightened vulnerability that may be explained by several factors. GI cancers often require multimodal, intensive treatment strategies, including surgery, chemotherapy, and/or radiation therapy, commonly resulting in high out-of-pocket costs and prolonged employment disruption [16, 17]. Moreover, GI cancer survivors may experience long-term health challenges such as ostomy care, nutritional deficiencies, or chronic gastrointestinal symptoms that elevate daily living expenses and limit their ability to fully resume employment [16, 18, 19]. Collectively, these challenges can perpetuate a cycle of financial strain and unmet needs. Qualitative evidence supports this interplay, with patients and providers describing situations in which limited resources force choices between medical care and essential expenses such as food and housing—effectively linking healthcare costs to other hardships [1, 3].

Importantly, even GI cancer survivors without reported financial hardship were more likely than controls to experience food insecurity and multiple HRSNs. This data suggest that structural factors beyond direct medical expenses, such as baseline poverty, disability, or weaker social support mechanisms contribute to more unmet social needs [20]. In contrast, non-GI cancer survivorship without financial hardship demonstrated little differentiation from the general US population. These findings are consistent with and build upon prior studies on financial hardship and social determinants in oncology. For instance, Xing et al. reported dramatically higher prevalence of food, housing, and transportation insecurity among survivors with financial hardship, while Hallgren et al. noted that financial hardship was associated with increased risk of household food insecurity, despite food insecurity being relatively uncommon in the overall survivor population [9, 12]. Of note, the current study adds to this body of literature by highlighting the impact of cancer-type heterogeneity and identifying GI cancer survivors as a subgroup of patients with particularly acute needs, potentially due to the well-documented high cost of GI cancer care and the intense symptom burden [21]. Notably, the co-occurrence of hardship and HRSNs may have important implications. Food and housing insecurity are not merely socioeconomic indicators but have been linked to adverse cancer outcomes, including increased emergency department visits and higher mortality [13, 22]. For instance, Hong et al. demonstrated that food-insecure survivors had greater all-cause mortality, underscoring that unmet basic needs directly influence survivorship and longevity [13]. Taken together, the findings reinforce the concept that comprehensive cancer survivorship care must address both financial strain and fundamental social needs.

Clinically and policy-wise, the current findings support standardized screening for medical financial hardship and HRSNs, particularly among GI cancer survivors, as this patient subgroup is at a high-risk. Unfortunately, systematic HRSN assessment remains uncommon in oncology, leaving many social needs undetected [23]. Effective interventions should combine detection with actionable, evidence-informed support resources [23, 24]. Financial toxicity can be addressed through financial navigation programs that assist patients in optimizing insurance coverage, negotiating medical bills, and accessing assistance programs, thereby reducing cost-related distress and potential treatment delays [25, 26]. For HRSNs, multicomponent, navigator- or community-health-worker–led models that concurrently address transportation, nutrition, and housing support are more likely to improve outcomes than single-need approaches, especially for survivors facing multiple, concurrent constraints [6, 12, 27]. Recent CMS reimbursement mechanisms for social risk screening create opportunities to integrated screening and resource alignment into routine oncology workflows [27, 28]. However, these policies warrant rigorous evaluation in cancer populations to determine effectiveness, scalability, and equity impacts; if effective, such policies may promote supportive care and reduce disparities among cancer survivors [29, 30].

Findings of the current study should be interpreted in light of certain limitations. NHIS is cross-sectional, capturing associations at one point and precluding causal inference. In addition, HRSNs and financial hardship can bidirectional, and the directional relationship could not be determined. NHIS also did not consistently capture time since diagnosis across survey years; in turn, survivorship duration could not be included in the adjusted analyses, which may have resulted in conservative estimates if the early post-diagnosis period (with higher treatment intensity and work disruption) was underrepresented. Cumulative hardship among long-term survivors could have also influenced observed associations. Because NHIS samples only community-dwelling survivors, high-mortality GI cancers (e.g., pancreatic and gastric) were likely underrepresented, which may underestimate hardship in the broader GI cancer population. Although we adjusted for age and conducted age-stratified analyses, residual confounding from unmeasured age-related factors (e.g., disability, fixed income, caregiving burden) may remain. Measures were self-reported, introducing possible misclassification or under-reporting due to stigma. Using the NHIS imputed income-to-poverty guideline ratio, GI cancer survivors were more likely to have household income ≤ 138% of the poverty guideline (21.1%, n = 453) than non-GI cancer survivors (16.7%, n = 2,576) and the general population (19.9%, n = 42,380) (p < 0.001); corresponding distributions across 139–200%, 201–400%, and > 400% were 13.9%, 33.6%, and 31.4% for GI survivors. Finally, pooling GI cancer types increased power but may mask heterogeneity; cancer stage and treatment were unavailable and may confound associations.

In conclusion, GI cancer survivors in the US experience a disproportionate burden of financial and social hardships compared with both non-GI cancer survivors and the general population. Medical financial hardship was strongly associated with unmet social needs, and this relationship was especially pronounced among GI cancer survivors. As the survivor population grows, addressing the dual, intertwined challenges of financial toxicity and HRSNs will be essential to improve survivorship outcomes, with GI cancer survivors representing a priority population for such interventions.

## Supplementary Information

Below is the link to the electronic supplementary material.

ESM 1 (DOCX  22.9 KB)

ESM 2 (TIF Supplementary FigureS1. Derivation of the analytic cohort and cancer survivor subgroups (NHIS 2013–2018; IPUMS NHIS extract). The flow diagram summarizes the creation of the adult analytic cohort from the initial IPUMS NHIS extract. Respondents were excluded if they were not part of the Sample Adult analytic file and/or were aged <18 years, yielding the final eligible adult cohort (n = 190,113). Within the final cohort, respondents were classified as having no cancer history (N=176,127) or as cancer survivors (Any cancer survivor= 1; n = 13,986), and cancer survivors were further categorized as GI cancer survivors (N =1,722) or non-GI cancer survivors (N= 12,264). All counts are unweighted. 58.0 KB)

## Data Availability

No datasets were generated or analysed during the current study.
