# Supplementary material for: Distinct financial and social hardships among gastrointestinal cancer survivors: a national survey analysis
Source: J Cancer Surviv. 2026 Mar 18;20(Suppl 2):1722–9. doi: 10.1007/s11764-026-02004-6 (PMC13744476; doi:10.1007/s11764-026-02004-6)
Supplement: Supplementary file 1 [file 11764_2026_2004_MOESM1_ESM.docx]

**Supplementary table S1.** Prevalence of health‑related social needs among cancer survivors, stratified by medical financial hardship status.

| **Outcome** | **Medical Financial Hardship** | | **p-value** |
| --- | --- | --- | --- |
|  | **Yes N (%)** | **No N (%)** |  |
| **Food Insecurity** |  |  | <0.001 |
| GI cancer | 148 (21.0) | 64 (4.8) |  |
| Non-GI cancer | 877 (17.2) | 354 (3.6) |  |
| **Housing insecurity** |  |  | <0.005 |
| GI cancer | 225 (35.6) | 114 (11.7) |  |
| Non-GI cancer | 1,554 (34.3) | 680 (8.6) |  |
| **Transport barrier** |  |  | <0.001 |
| GI cancer | 46 (5.0) | 24 (1.9) |  |
| Non-GI cancer | 252 (4.8) | 156 (1.9) |  |
| **Any HRSN** |  |  | <0.005 |
| GI cancer | 296 (45.1) | 155 (14.9) |  |
| Non-GI cancer | 1,897 (41.1 | 962 (11.8) |  |
| **Multiple HRSNs (≥ 2)** |  |  | <0.001 |
| GI cancer | 105 (15.1) | 42 (3.2) |  |
| Non-GI cancer | 678 (13.3) | 208 (2.2) |  |

_* Data represent survey-weighted estimates. Categorical variables are reported with proportions (%) and continuous variables are reported using median and interquartile range._

**Supplementary Table S2. Interaction of Cancer Survivor Subgroup and Medical Financial Hardship on Health‑Related Social Needs**
(Reference group = U.S. general population without financial hardship).

| **Outcome** | **Interaction aOR**  **Non‑GI (95 % CI)** | **p‑value** | **Interaction aOR**  **GI (95 % CI)** | **p‑value** |
| --- | --- | --- | --- | --- |
| **Food insecurity** | 1.20 (0.97 – 1.48) | 0.087 | 1.02 (0.61 – 1.68) | 0.952 |
| **Housing insecurity** | 1.48 (1.26 – 1.73) | <0.001 | 1.23 (0.81 – 1.87) | 0.334 |
| **Transportation barrier** | 1.08 (0.75 – 1.57) | 0.667 | 0.85 (0.41 – 1.76) | 0.655 |
| **Any HRSN** | 1.35 (1.15 – 1.57) | <0.001 | 1.24 (0.84 – 1.83) | 0.285 |
| **Multiple HRSNs (≥ 2)** | 1.34 (1.04 – 1.73) | 0.026 | 0.96 (0.55 – 1.67) | 0.891 |

_Adjusted odds ratios (aOR) and 95 % confidence intervals (CI) for the interaction between survivor subgroup (Non‑GI vs. GI cancer survivors) and medical financial hardship (yes vs. no) on each health‑related social need. Estimates derive from pooled, survey‑weighted multivariable logistic regressions with an ib0.med_hardship##ib0.cohort term (reference = U.S. general population without hardship). Models adjust for age category, sex, race/ethnicity, education, marital status, income category, insurance status, and geographic region. P‑values < 0.05 denote statistical significance._

**Supplementary Table S3. Age-stratified interaction of cancer survivor subgroup and medical financial hardship on HRSNs (Reference group = General U.S. population without medical financial hardship, within each age stratum).**

| **Outcome** | **Age Category** | **Interaction aOR non-GI (95% CI)** | **p-value** | **Interaction aOR GI (95% CI)** | **p-value** |
| --- | --- | --- | --- | --- | --- |
| **Food insecurity** | 18–64 | 1.14 (0.88–1.46) | 0.320 | 0.80 (0.43–1.50) | 0.485 |
|  | 65–74 | 0.95 (0.53–1.72) | 0.877 | 0.75 (0.21–2.61) | 0.649 |
|  | ≥75 | 1.53 (0.74–3.17) | 0.248 | 2.46 (0.64–9.48) | 0.191 |
| **Housing insecurity** | 18–64 | 1.40 (1.15–1.71) | 0.001 | 1.05 (0.59–1.88) | 0.873 |
|  | 65–74 | 1.24 (0.81–1.89) | 0.329 | 0.72 (0.30–1.71) | 0.454 |
|  | ≥75 | 0.96 (0.63–1.46) | 0.840 | 1.51 (0.60–3.83) | 0.383 |
| **Transportation barrier** | 18–64 | 0.93 (0.58–1.49) | 0.750 | 0.82 (0.28–2.45) | 0.727 |
|  | 65–74 | 1.69 (0.76–3.72) | 0.195 | 0.58 (0.15–2.29) | 0.435 |
|  | ≥75 | 0.94 (0.39–2.27) | 0.889 | 1.12 (0.23–5.44) | 0.889 |
| **Any HRSN** | 18–64 | 1.25 (1.03–1.52) | 0.022 | 0.99 (0.57–1.71) | 0.978 |
|  | 65–74 | 1.24 (0.86–1.80) | 0.256 | 0.76 (0.33–1.72) | 0.505 |
|  | ≥75 | 1.05 (0.71–1.56) | 0.814 | 1.83 (0.76–4.40) | 0.177 |
| **Multiple HRSNs (≥2)** | 18–64 | 1.31 (0.97–1.76) | 0.081 | 0.93 (0.50–1.76) | 0.835 |
|  | 65–74 | 0.98 (0.49–1.98) | 0.965 | 0.43 (0.11–1.62) | 0.212 |
|  | ≥75 | 1.25 (0.48–3.27) | 0.653 | 1.57 (0.26–9.46) | 0.619 |

_Adjusted odds ratios (aORs) and 95% confidence intervals (CIs) for the interaction between cohort (Non-GI vs. General; GI vs. General) and medical financial hardship (yes vs. no) within each age stratum, estimated using pooled survey-weighted multivariable logistic regression. Models adjust for sex, race/ethnicity, education, marital status, income category, insurance status, and region._

**Supplemental Table S4:** Sensitivity analyses among pancreatic or gastric cancer survivors: association between medical financial hardship and health-related social needs (NHIS 2013–2018)

| **Outcome (panc/gast survivors)** | **Subgroup size used in**  **model (unweighted)** | **OR for med_hardship=1 vs 0**  **(95% CI)** | **p-value** |
| --- | --- | --- | --- |
| **Any HRSN** | 165 | **3.58** (1.05–12.25) | 0.042 |
| **Food insecurity** | 169 | **2.46** (0.60–10.13) | 0.211 |
| **Housing insecurity** | 169 | **2.38** (0.97–5.82) | 0.057 |
| **Multiple HRSN** | 169 | **1.70** (0.40–7.28) | 0.474 |

_Abbreviations: NHIS, National Health Interview Survey; HRSN, health-related social needs; OR, odds ratio; CI, confidence interval. The analytic subgroup included respondents with a history of pancreatic or stomach (gastric) cancer, defined as cnpanc=2 and/or cnstom=2 among cancer survivors (any_cancer=1). All models were survey-weighted using NHIS design variables. Reported ORs represent the association of medical financial hardship (yes vs no) with each outcome._
